# BCL3 expression is strongly associated with the occurrence of breast cancer relapse under tamoxifen treatment in a retrospective cohort study

**DOI:** 10.1007/s00428-021-03238-8

**Published:** 2022-01-12

**Authors:** Piotr Czapiewski, Maximilian Cornelius, Roland Hartig, Thomas Kalinski, Johannes Haybaeck, Angela Dittmer, Jürgen Dittmer, Atanas Ignatov, Norbert Nass

**Affiliations:** 1 Department of Pathology, Medical Faculty, Otto-Von-Guericke University Magdeburg, Leipziger Str. 44, 39120 Magdeburg, Germany; 2 Department of Pathology, Dessau Medical Center, Auenweg 38, 06847 Dessau, Germany; 3 Institute for Molecular and Clinical Immunology, Medical Faculty, Otto-Von-Guericke University Magdeburg, Leipziger Str.44, 39120 Magdeburg, Germany; 4 Multi-Parametric Bioimaging and Cytometry Platform, Medical Faculty, Otto-Von-Guericke University Magdeburg, Leipziger Str.44, 39120 Magdeburg, Germany; 5 Diagnostic & Research Center for Molecular BioMedicine, Institute of Pathology, Medical University Graz, Neue Stiftingtalstrasse 6, 8010 Graz, Austria; 6 Institute of Pathology, Neuropathology and Molecular Pathology, Medical University of Innsbruck, Müllerstraße 44, 6020 Innsbruck, Austria; 7 Clinic for Gynecology, Martin-Luther University, Halle-Wittenberg Ernst-Grube-Straße 40, 06120 Halle (Saale), Germany; 8 Department of Obstetrics and Gynecology, Otto Von Guericke University Magdeburg, Gerhart-Hauptmann Str. 35, 39108 Magdeburg, Germany; 9 Dessau Medical Center, Department for Internal Medicine I, Auenweg 38, 06847 Dessau, Germany

**Keywords:** Breast cancer; Tamoxifen, BCL3; Immunohistochemistry; Biomarker, Survival

## Abstract

Patients with estrogen receptor positive breast cancer are usually receiving an anti-estrogen therapy by either aromatase inhibitors or selective estrogen receptor mediators such as tamoxifen. Nevertheless, acquired resistance to tamoxifen under treatment frequently hampers therapy. One proposed explanation for this phenomenon is the interaction of the tumor cells with cells of the tumor microenvironment via the Insulin-like growth factor RNA binding protein 5/B-cell lymphoma 3 (IGFBP5/BCL3) axis. Here we investigated whether a high expression of BCL3 either cytoplasmic or nuclear is associated with the occurrence of a relapse under anti-estrogen therapy in patients. Formaldehyde-fixed, paraffin-embedded samples of 180 breast cancer patients were analyzed for BCL3 expression by immunohistochemistry. An immunoreactive score (IRS) was calculated from staining intensity in cytoplasm and nucleus as well as the percentage of positive tumor cells. These scores were correlated with clinico-pathological parameters using cross-tabulation analysis and patients’ relapse free and overall survival by Kaplan–Meier analysis and Cox regression. A tamoxifen-adapted MCF-7 derived cell line was investigated for BCL3 localization by immunofluorescence. The cytosolic BCL3-IRS significantly correlated with the proliferation marker Ki-67, and with the occurrence of a relapse under tamoxifen treatment. Nuclear score correlated only with tamoxifen-relapse. In survival analysis, both scores were highly significant prognostic factors for relapse free, but not for overall survival. This was especially obvious for estrogen receptor positive and HER2/NEU negative cases as well as lobular breast cancer. Tamoxifen-treated, but not aromatase-treated patients had a poor survival when BCL3 scores were high. A tamoxifen adapted cell line exhibited a reduced expression and mainly nuclear localization of BCL3, compared to the parental estrogen receptor positive cell-line MCF-7. Altogether, these data strongly support a function of BCL3 in tamoxifen resistance and its potential use as a predictive biomarker for tamoxifen resistance.

**Supplementary Information:**

The online version contains supplementary material available at 10.1007/s00428-021-03238-8.

## Background

Breast cancer (BC) is the most frequent neoplasia in women worldwide. Although it has an, on average, good outcome, some subtypes of this heterogeneous disease still impose a problem in the clinic [1]. Clinically, BC is classified mainly by immuno-histochemistry (IHC) according to the estrogen- and progesterone receptor status, the increased expression of the epidermal growth factor receptor HER2/NEU (erbb2), and the proliferation rate as determined by the Ki-67 status. Estrogen receptor (ER) positive cases are treated with anti-endocrine therapies. For this purpose, either selective estrogen receptor modulators (SERMs) such as tamoxifen or selective estrogen receptor degraders (SERDs) such as fulvestrant or inhibitors of estrogen biosynthesis such as anastrozole [2] are in clinical use. In premenopausal patients, tamoxifen seems more effective than aromatase inhibitors, although about 25% of the patients experience a relapse under this therapy [3]. For these cases, a predictive biomarker would be supportive for choosing an alternative therapy before relapse occurs. Tamoxifen resistance can be caused by several mechanisms. Firstly, mutations in the ER can cause constitutive activity [4] and such alterations are enriched during endocrine therapy. ESR1 mutations seem more important for aromatase inhibitor treatment, compared to tamoxifen therapy [5]. Secondly, tamoxifen resistance can be acquired over time, which comprises a switch from ER-dependent proliferation to other mechanisms such as epidermal growth factor- (EGF) or insulin-like growth factor- (IGF)- or NF-kb-signaling [6]. Also, estrogen signaling via alternative, membrane bound estrogen receptors such as GPER1 [7] and splice forms of the ER [8] are possible mechanisms. Another important factor is the influence of the tumor microenvironment [9].

The B-cell-lymphoma-3 (BCL3) protein has first been identified as over-expressed protein in hematological cancers. In these entities, its oncogenic activity is due to its influence on p53 as well as cyclinD1 expression [10, 11]. BCL3 is part of the NF-kB transcriptional regulatory system, belongs to the IκB family, and interacts with the NF-kB homodimers (p50, p52) as a transcriptional coactivator [12]. However, it can also act independently of NF-kB on proliferation, metastasis, and apoptosis [13]. In the cytosol, BCL3 is usually un-phosphorylated and has similar inhibitory functions as other IkB proteins on p50 (NFKB1) and p52 (NFKB2). Upon activation by e.g. erythropoietin or granulocyte–macrophage colony-stimulating factor BCL3 translocates to the nucleus [14].

This translocation and nuclear activity depends on ubiquitinylation [15] and phosphorylation by AKT, ERK, or IKK1/2 [16].

Consequently, BCL3 was found in cancer cells in the cyctosol as well as in the nucleus [17]. In breast cancer, BCL3 has been found to be induced under estrogen depletion [18]; it promotes proliferation of the TNBC cell line MDA-MB-468 [19], regulates TGFβ-signaling during breast cancer metastasis [20], and promotes metastasis in erbb2-positive tumors [21]. Interestingly, nuclear BCL3 is upregulated in MCF-7 BC cells, in response to the presence of cancer-associated fibroblasts or mesenchymal stem cells via downregulation of IGFBP5 and this is important for desensitizing BC cells to fulvestrant [22].

However, the prognostic potential of BCL3 for endocrine therapy has not been investigated yet. We here investigated whether BCL3 determined by IHC has a potential as predictive biomarker for tamoxifen resistance.

## Materials and methods

### Patients and data analysis

BC patients of the Otto von Guericke University Magdeburg were recruited from 1999 to 2009 [23]. The ethics commission of this University approved the study (file number AKZ 114/13). Follow-up data were obtained from the files of the Clinic of Obstetrics and Gynecology and pathological diagnosis from the records of the Institute of Pathology. This patient collective has been investigated in several projects before; thus, not all paraffin blocks contained still enough material for an immuno-histochemical staining. As a result, 180 samples could be evaluated for BCL3 expression. Pathological data on receptassessed at the time of diagnosisor expression, TNM scoring and grading were assessed at the time of diagnosis [23]. Statistical analysis was performed with SPSS vers. 19 (IBM). A statistical significance of *p* < 0.1 was considered as; *p* < 0.05 values were considered statistically significant.

#### IHC

Formalin-fixed, paraffin-embedded tumor samples were sectioned (2 µm), deparaffinized by xylol, and antigen retrieval was achieved in CC1 mild buffer. All slides were stained using an automated staining system (Benchmark Ultra, Ventana). The primary antibody (abcam 125,217, 1/200) was added in Ventana antibody dilution buffer. Detection was performed using the Ventana Ultraview DAB staining reagents. For establishing the demasking and staining conditions, sections of tonsillar tissue were used and Western blots with breast cancer cell line proteins were performed. Nuclei were counterstained using hematoxylin. The stained sections were evaluated for staining intensity (0 = no, 1 = weak, 2 = intermediate, and 3 = strong intensity) as well as percentage of positive tumor cells (in 10% intervals) conjointly by PC and NN, using a light microscope equipped with a digital camera. Both parameters were multiplied and then divided by ten to obtain an immuno-reactive score (IRS). Both scores were determined for nuclear and cytoplasmic signals separately.

### Indirect immunofluorescence

MCF-7 cells and a tamoxifen adapted MCF-7 cell line [24, 25] were used for these studies. Cells were seeded onto glass slides (Sarstedt), fixed using ice cold methanol followed by acetone (− 20 °C) for 5 min each. Slides were blocked by normal goat serum in TRIS-buffered saline (TBS) supplemented with 0.1% Triton X-100. Primary antibodies were added in TBS/Tween 20 (0.05%) incubated at 4 °C overnight and detected after three washing steps (TBS) using dylight 488 secondary antibody (Thermo-Fisher). Nuclei were counterstained using a propidium iodide containing embedding medium (Vectashield, Vector Laboratories). Slides were visualized using an inverted Confocal Microscope System Leica SP8 (Leica Mannheim, Germany) equipped with a Plan Apo 63x/1.4 oil objective and controlled by the LASX software (Leica). To avoid bleed-through between the different spectral channels, sequential unidirectional scanning was performed at 600 Hz using the following settings: sequence 1: excitation 488 nm, emission 500–549 nm; sequence 2: excitation 561 nm, emission 606–665 nm combined with transmitted light detection. Sequences were altered between lines. Voxel size was adjusted to 92 nm × 92 nm × 230 nm (dx, dy, dz) to fit to Nyquist theorem. Images of the individual channels were pseudo colored: propidium iodide (excitation 561 nm) in red and DyLight488 (excitation 488 nm) in green. Single planes out of the data stacks were analyzed using ImageJ software.

### Western blotting

For Western blotting, proteins were separated on a 12% denaturing poly-acrylamide gel and transferred to nitrocellulose by semi-dry blotting [26]. Antigen detection was performed using the same antibodies as applied for histochemistry diluted in TBS containing BSA (2%) and NP-40 (0.2%). After washing and incubation with a peroxidase coupled secondary antibody (Jackson-Laboratory) and three washing steps, the signal was detected using enhanced chemiluminescence (Millipore) by a chemostar imager (INTAS, Goettingen, Germany).

### Database analysis

mRNA data were either obtained via cBioPortal [27] from the METABRIC dataset and reformatted for use in SPSS or analyzed directly on the website (GEPIA2) [28].

## Results

### Analysis of BCL3 expression in tamoxifen adapted cell lines (MCF-7-TamR)

**Fig. 1:**
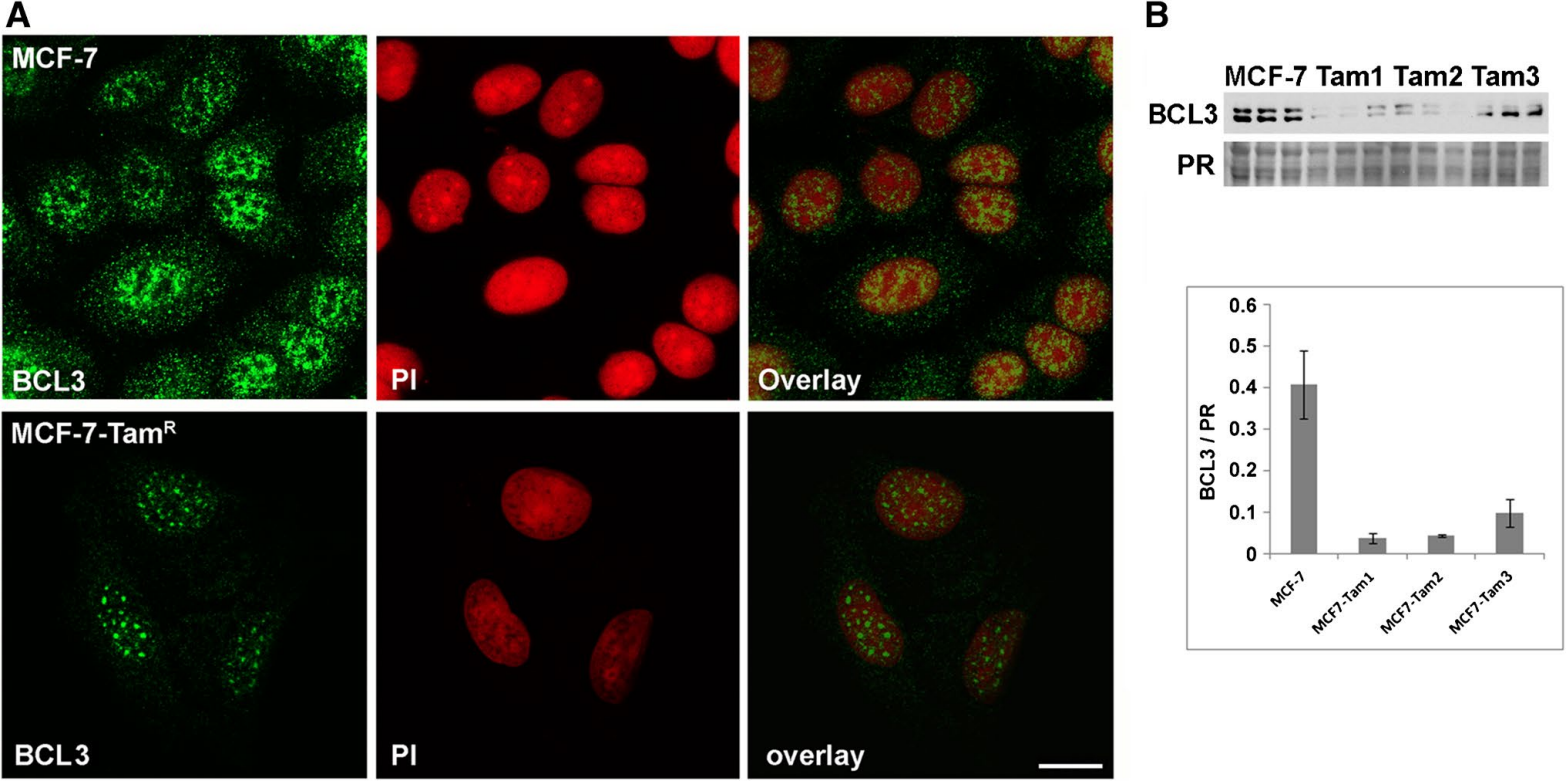
**A** Indirect immunofluorescent staining of BCL3 in MCF-7 and MCF7-TamR cells. Cells were stained using the BCL3 antibody and a secondary fluorescent antibody. The nuclei were counter-stained using propidium iodine (PI). Images were obtained using a laser scanning microscope as described in “Materials and methods.” The scale bar represents 50 µm. **B** Western blot analysis of BCL3 in protein extracts of MCF-7 compared to three Tam-adapted cell lines (TamR) derived from this cell line. BCL3 Western blot signals as well as poinceau red protein stain (PR) are shown. The bar graph indicates the BCL3 signal normalized to the PR staining result averaged for each cell line with standard deviation

In addition to earlier studies on anti-estrogen resistance and the effect of cancer-associated fibroblasts, we initially investigated the BCL3 expression in our model for acquired tamoxifen resistance. In this model, the luminal A cell line MCF-7 was adapted to 4OH-tamoxifen for at least 12 weeks [23, 24]. Here, we were particularly interested whether BCL3 localization and abundance has changed. In our cDNA array data, *BCL3* mRNA was not significantly altered during tamoxifen adaption of MCF-7 [24]. However, Western blots of three independently generated MCF7-TamR lines detected decreased amounts of BCL3 protein (Fig. 1) although with significant variation. In immunofluorescence analysis, MCF-7-TamR cells showed BCL3 mainly localized to the nucleus in a dotted appearance compared to MCF-7 (Fig. 1). The signal ratio cytosol to nucleus was determined to be 0.48 ± 0.14 and 0.37 ± 0.13 for MCF-7 and MCF-7-TamR, respectively (*p* = 0.014).

### Distribution of BCL3 abundance by immunohistochemistry in the patient cohort

**Fig. 2:**
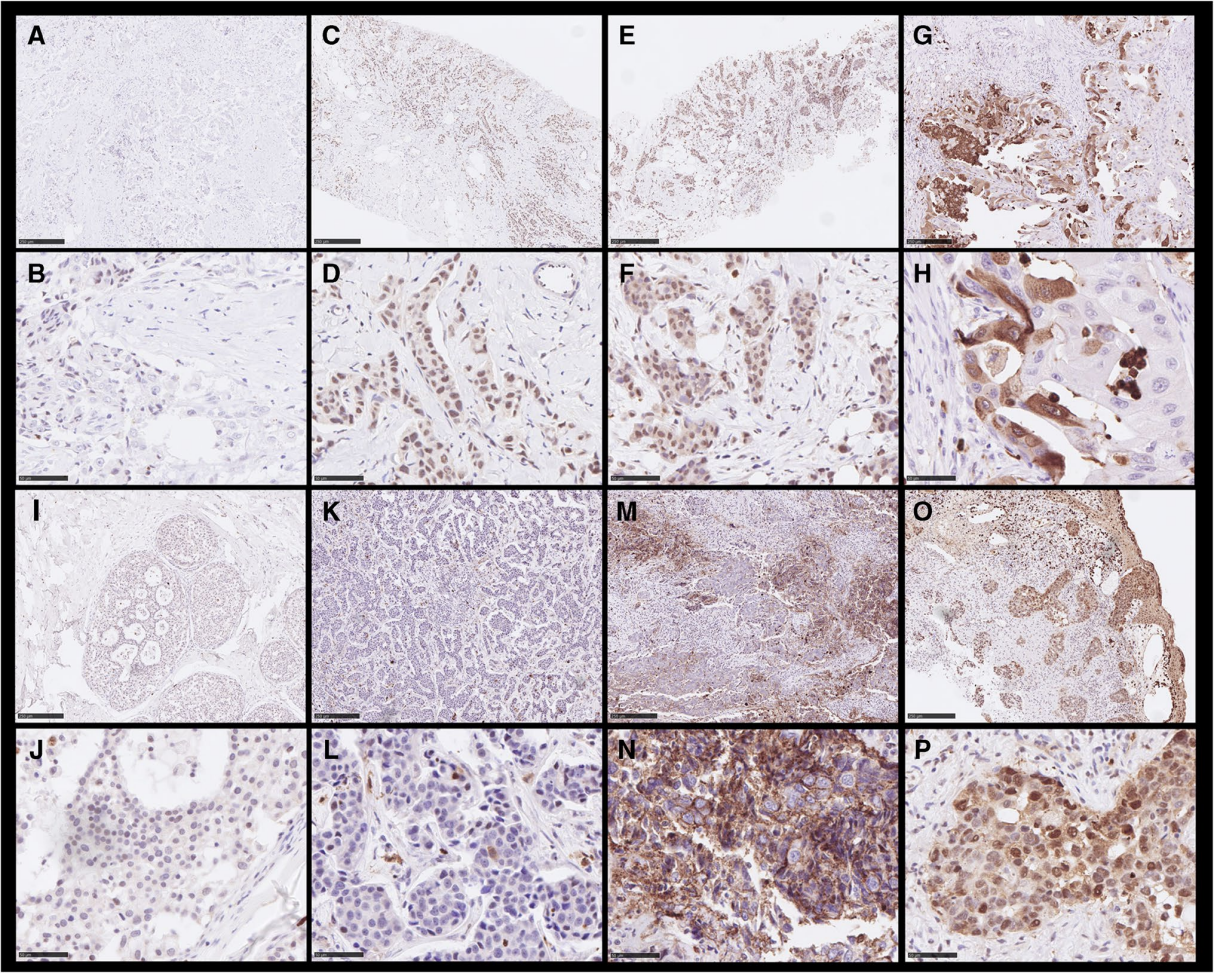
Representative results of the IHC of BCL3 in BC samples. Scale bars indicate 50 or 250 µm, respectively. **A**, **C**, **E**, **G**, **I**, **K**, **M**, and **O** show low magnification; **B**, **D**, **F**, **H**, **J**, **L**, **N**, and **P** high magnification. Intensity- and %-scores: **A**, **B**: cyt. 0, 0 nucl. 1, 3 (I, %); **I**, **J** (cibriform DCIS): cyt. 0, 0 nucl. 1, 15 (I, %); **C**, **D**: cyt. 1, 90 nucl. 3, 95 (I, %); **K**, **L**: cyt. 1, 40 nucl. 1, 3 (I, %); **E**, **F**: cyt. 2, 90 nucl. 3, 80 (I, %); **M**, **N**: cyt. 2, 30 nucl. 0, 0 (I, %); **G**, **H**: cyt. 3, 40 nucl. 0, 0 (I, %); **O**, **P**: cyt. 3 100 nucl. 3, 70 (I, %)

**Fig. 3:**
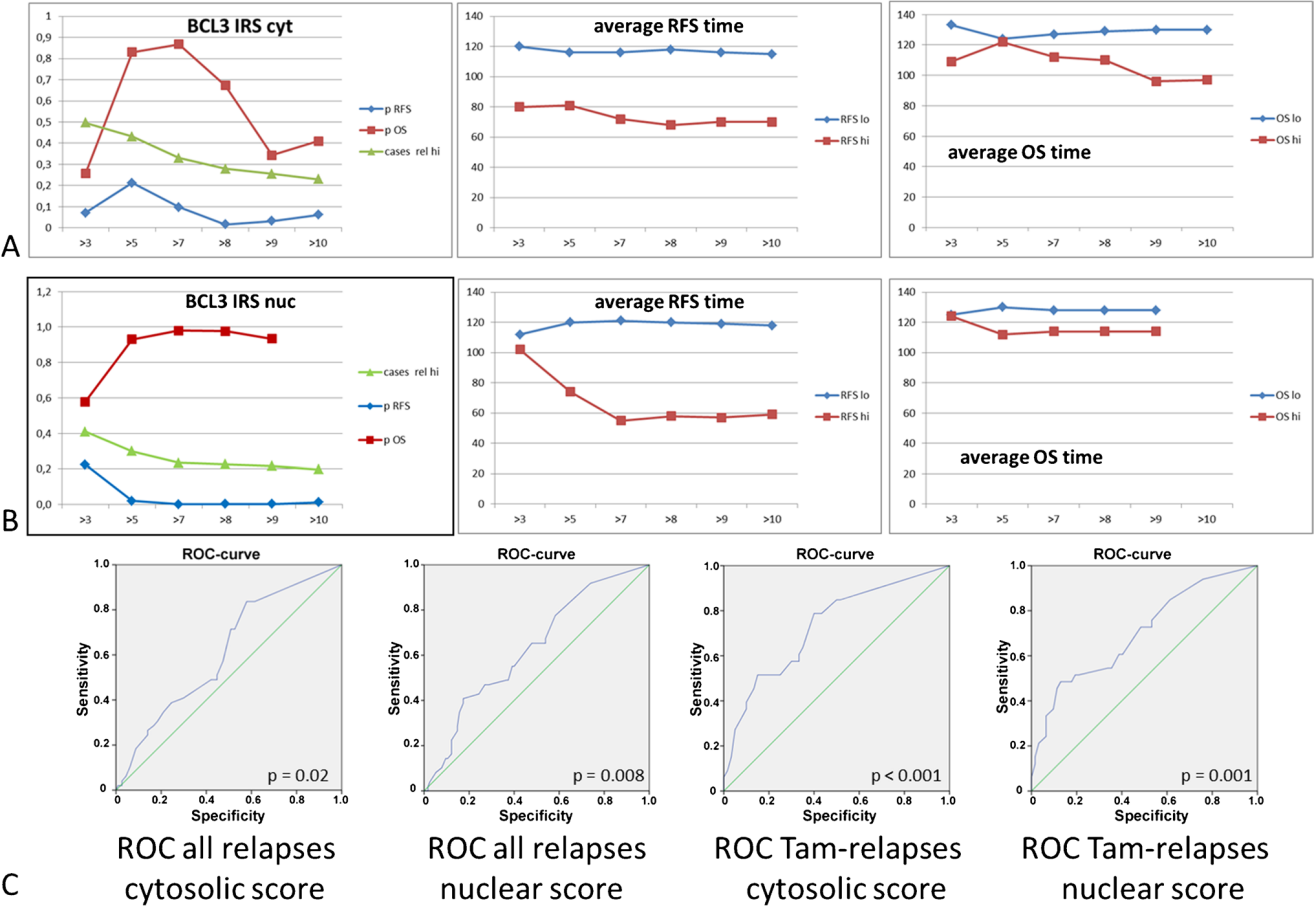
Determination of a cut-off value for BCL3 IRS based on Kaplan–Meier survival analysis. **A** Data for cytosolic BLC3. Log-rank p for RFS, OS, the relative number of BCL3 high cases and the average relapse-free and overall-survival time depending on the cut-off value are shown. **B** Data for nuclear BCL3 IRS are shown as described for **A**. **C** Receiver-operator-curves (ROC) for cytosolic and nuclear IRS and relapse-free survival for all cases and tamoxifen-treated cases

We then stained paraffin-embedded tissue of our breast cancer cohort for BCL3 by immunohistochemistry. Here, we observed a specific staining of BCL3 in tumor cells in both, the cytoplasmic and nuclear compartment but this varied between the specimens (Fig. 2). As consequence, we scored the IHC signal for nucleus and cytosol separately. A cut-off value for the immuno-reactive score (IRS) was determined separately for nuclear and cytosolic staining by optimizing the log-rank *p*-value in Kaplan–Meier survival analysis and using the receiver operator curve (ROC) for relapse-free survival. A cut-off value was set to IRS > 8 for both localizations (Fig. 3). The distribution of high and low abundance of BCL3 according to clinico-pathological parameters is summarized in Table 1. Overall, high cytoplasmic BCL3 was detected in 31.7% of all cases, whereas high nuclear BCL3 was found for 22.8% of the tumors. There was an intermediate correlation of cytosolic and nuclear BCL3 IRS (Spearman’s rho = 0.24, *p* = 0.001). Only 16% of low cytoplasmic BCL3 cases had high nuclear BCL3 and 42.1% of high cytoplasmic BCL3 tumors exhibited also high nuclear BCL3 IRS (Fisher’s exact test *p* < 0.001). There was a weak negative association of cytoplasmic score (p = 0.063) with ER-status. A positive association of the cytoplasmic score was found for tumor size (T > 2, *p* = 0.059) and Ki-67-status (*p* = 0.001). Most interestingly both the high cytoplasmic and high nuclear BCL3 IRS correlated strongly with the appearance of a relapse under tamoxifen therapy (*p* < 0.001). However, in contrast to the cytosolic BCL3-IRS, nuclear BCL3-status did not correlate with the other factors tested.

**Table 1:** Cohort characteristics with respect to BCL3 IRS and clinico-pathological parameters. Significance was determined by two sided Fisher’s exact test or ordinal by ordinal correlation (§). *ER-positive cases only

Tumor type	number	BCL3-IRS_cyt_ lo/hi > 8 (high %)	Significance	number	BCL3-IRS_nuc_ lo/hi > 8 (high %)	Significance
All	180	123/57 (31.7%)		180	139/41 (22.8%)	
Menopause			0.839			0.657
Pre-	35	24/11 (31.4%)		35	28/7 (20.0%)	
Post-	127	89/38 (29.9%)		127	95/32 (25.2%)	
ER			0.063			0.826
Negative	35	20/15 (42.9%)		35	26/9 (25.7%)	
Positive	126	93/33 (26.2%)		126	96/30 (23.8%)	
PR			0.487			0.459
Negative	69	46/23 (24.0%)		69	50/19 (27.5%)	
Positive	92	67/25 (27.2%)		92	72/20 (21.7%)	
HER2			0.836			0.181
Negative	123	88/35 (28.5%)		123	97/26 (21.1%)	
Positive	36	25/11 (30.6%)		36	24/12 (33.3%)	
TNBC			1.000			0.599
No	137	97/40 (29.2%)		137	103/34 (24.8%)	
Yes	22	16/6 (27.3%)		22	18/4 (18.2%)	
Lymph node			0.285			0.191
Negative	95	64/31 (32.6%)		95	68/27 (28.4%)	
Positive	64	49/15 (23.4%)		64	52/12 (18.8%)	
Grading			0.257 ^§^			0.190 ^§^
1	19	13/6 (31.6%)		19	16/3 (15.8%)	
2	92	69/23 (25.0%)		92	71/21 (22.8%)	
3	50	31/19 (38.0%)		50	35/15 (30.0%)	
Histology			0.244			0.847
Ductal	129	91/38 (29.5%)		129	98/31 (24.0%)	
Lobular	23	19/4 (17.4%)		23	18/5 (21.7%)	
Other	6	3/3 (50.0%)		6	4/2 (33.3%)	
T > 2			0.059			0.462
No	76	59/17 (22.4%)		76	60/16 (21.1%)	
Yes	85	54/31 (36.5%)		85	62/23 (27.1%)	
Chemo-therapy			0.604			0.712
No	79	54/25 (31.7%)		97	59/20 (20.6%)	
Yes	81	59/22 (27.2%)		81	63/18 (22.2%)	
Radio-therapy			0.586			0.432
No	52	35/17 (32.7%)		52	37/15 (28.8%)	
Yes	109	78/31 (28.4%)		109	85/24 (22.0%)	
Endocrine therapy			0.438			0.841
None	29	18/11 (37.9%)		29	23/6 (20.7%)	
Tamoxifen	86	64/22 (25.6%)		86	65/21 (24.4%)	
Aromatase inhibitor	45	31/14 (31.1%)		45	33/12 (26.7%)	
Tam-relapse*			0.001			0.001
No	55	49/6 (10.9%)		55	49/6 (10.9%)	
Yes	25	13/12 (48.0%)		25	13/12 (48.0%)	
Ki-67			0.001			0.187
0–1	102	80/22 (21.6%)		102	82/20 (19.6%)	
2–3	64	34/30 (46.9%)		64	45/19 (29.7%)	

### Survival analysis

**Fig. 4:**
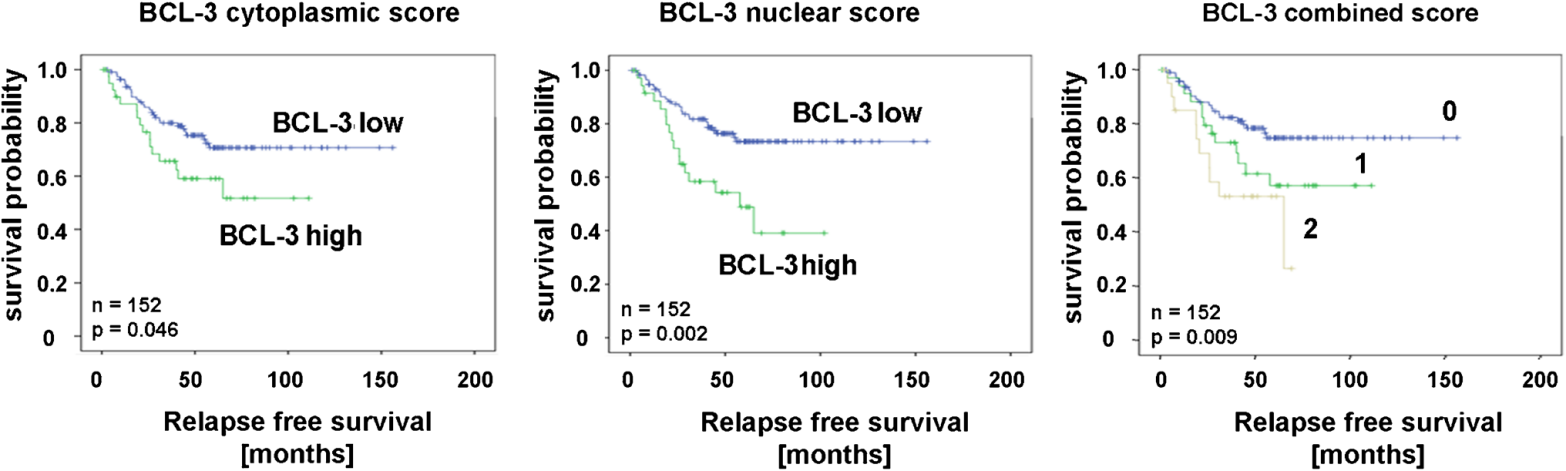
Kaplan–Meier plots for relapse-free survival (RFS) depending on BCL3-IRS_nuc_ and BCL3-IRS_cyt_ or a combined score. (0: negative in both locations; 1: positive in one location; 2: positive in both locations)

**Fig. 5:**
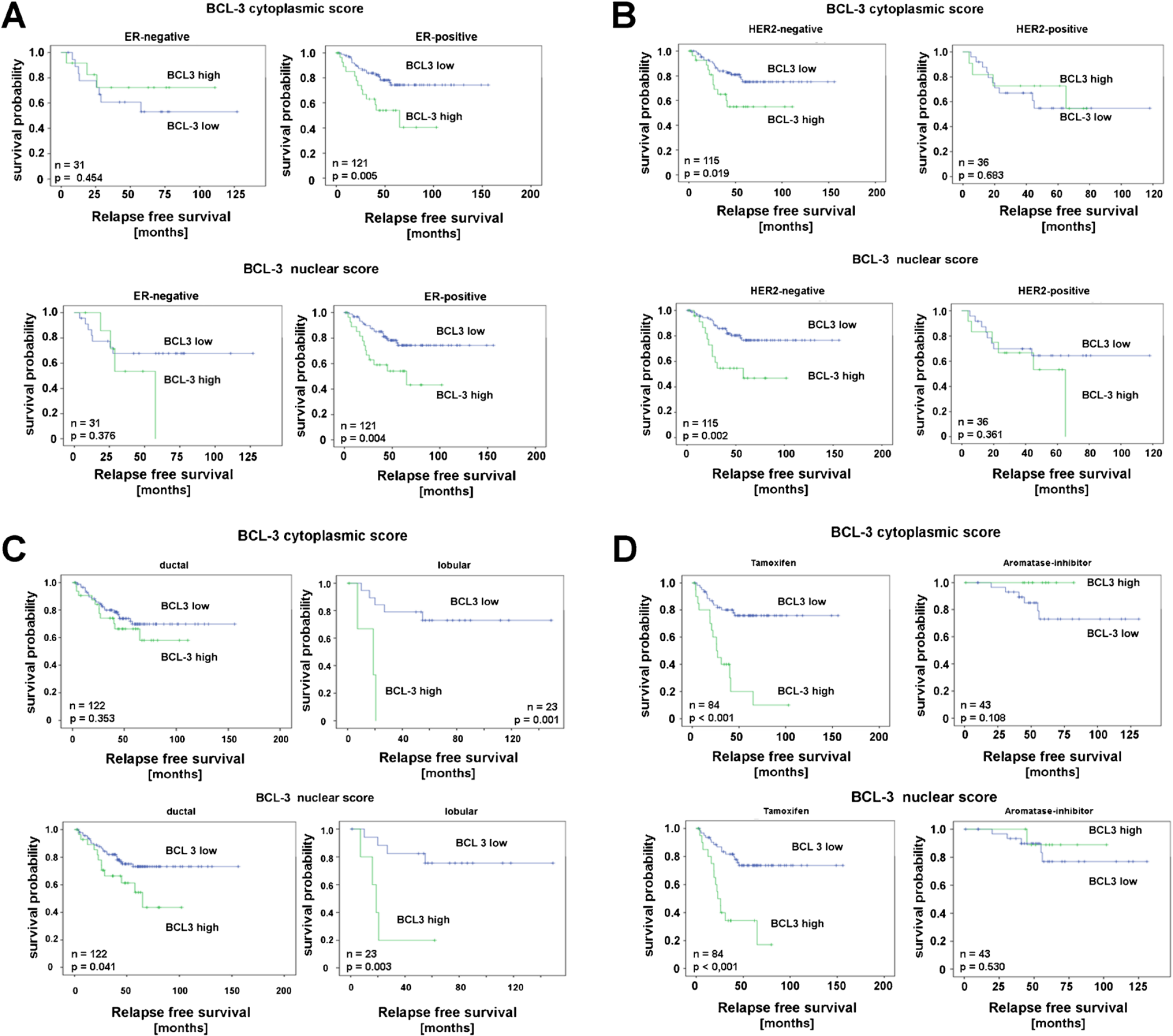
Kaplan–Meier plots for relapse-free survival depending on BCL3 nuclear and cytoplasmic score stratified for estrogen status (**A**), HER2 status (**B**), ductal and lobular histology (**C**), and treatment with tamoxifen or aromatase inhibitor (**D**)

We next evaluated the significance of BCL3-IRS for survival. Both high cytoplasmic and nuclear BCL3 IRS (> 8) were significantly associated with poor relapse-free survival (RFS, Fig. 3 and Fig. 4A) but not with overall survival. In cases that were high for BCL3 in both localizations, the correlation to RFS was even more pronounced (Fig. 4A). Concerning breast cancer subgroups, both scores were significant for tamoxifen treatment, lobular histology, G2, ER + , PR + , HER2-, Ki-67-low, and cases not treated by chemotherapy. Only the nuclear score was significantly correlated with RFS in post-menopausal cases, ductal histology, larger tumors and treatment by radiotherapy and chemotherapy (Fig. 5, Table 2). Interestingly, in aromatase inhibitor–treated cases, BCL3 IRS was not significant. Notably, the Kaplan–Meier curve for cases with low BCL3-IRS for aromatase inhibitor (AI)–treated patients was above the curve for BCL3-IRS high cases, suggesting a better response to this drug. When we restricted the Kaplan–Meier analysis to ER-negative cases, similar results were found (Table 2, Fig. 4A).

**Table 2:** Mean survival for breast cancer subclasses stratified for BCL3 high and low in cytosol and nucleus. Survival data were analyzed by the Kaplan–Meier method and log rank *p* is given. n.a. *Mean survival was not available when all cases were censored. ^§^Analysis restricted to ER-positive cases

	Cytoplasmic BCL3				Nuclear BCL3			
	> 8	Mean survival	SEM	*p*	> 8	Mean survival	SEM	*p*
All cases	Low	118.6	6.0	0.046	Low	121.6	5.8	0.002
	High	70.7	7.7		High	59.0	7.2	
Premenopausal	Low	112.6	12.7	0.533	Low	115.5	11.8	0.196
	High	68.8	15.1		High	50.6	12.5	
Postmenopausal	Low	118.8	6.8	0.057	Low	122.0	6.5	0.006
	High	70.0	8.6		High	58.6	8.0	
Ductal	Low	n.a. *		0.353	Low	n.a. *		0.041
	High	n.a. *			High	n.a. *		
Lobular	Low	n.a. *		0.001	Low	n.a. *		0.003
	High	n.a. *			High	n.a. *		
Other	Low	n.a. *		0.564	Low	n.a. *		0.317
	High	n.a. *			High	n.a. *		
T < 2	Low	128.6	7.4	0.677	Low	132.8	7.1	0.087
	High	82.8	10.2		High	72.0	10.8	
T > 2	Low	91.8	7.6	0.112	Low	94.2	7.1	0.025
	High	62.4	9.4		High	44.2	6.2	
N0	Low	128.1	7.2	.207	Low	133.3	6.6	0.006
	High	77.9	9.6		High	54.4	6.1	
N1	Low	92.0	7.8	0.146	Low	93.7	7.6	0.046
	High	47.3	9.0		High	49.5	12.3	
G1	Low	113.5	7.2	0.536	Low	n.a. *		0.552
	High	57.3	5.0		High	n.a. *		
G2	Low	121.7	7.4	0.009	Low	n.a. *		< 0.001
	High	55.6	10.6		High	n.a. *		
G3	Low	82.9	10.3	0.975	Low	n.a. *		0.665
	High	73.4	11.6		High	n.a. *		
ER-neg	Low	78.8	12.7	0.454	Low	90.8	11.3	0.376
	High	84.8	13.0		High	42.4	7.6	
ER-pos	Low	123.8	6.3	0.005	Low	123.8	6.3	0.004
	High	60.5	8.4		High	60.4	8.2	
PR-neg	Low	93.5	8.2	0.740	Low	98.4	7.6	0.108
	High	75.7	10.3		High	55.8	10.3	
PR-pos	Low	124.6	7.4	0.017	Low	125.2	7.3	0.007
	High	64.5	9.6		High	50.1	7.4	
HER2-neg	Low	125.3	6.4	0.019	Low	126.8	6.1	0.002
	High	72.1	8.9		High	61.8	8.6	
HER2-pos	Low	74.9	10.3	0.683	Low	82.6	10.2	0.361
	High	57.0	9.0		High	45.0	7.8	
No TNBC	Low	n.a. *		0.024	Low	123.8	6.0	0.003
	High	n.a. *			High	61.0	7.7	
TNBC	Low	n.a. *		0.357	Low	92.6	14.3	0.280
	High	n.a. *			High	43.3	14.5	
Radiotherapy no	Low	108.7	7.5	0.107	Low	108.6	7.6	0.133
	High	67.5	12.3		High	56.0	8.7	
Radiotherapy yes	Low	114.2	7.7	0.180	Low	118.6	7.2	0.008
	High	69.1	9.4		High	55.8	8.7	
Ki-67 < 2	Low	129.9	6.3	0.012	Low	133.5	5.9	< 0.001
	High	59.6	10.6		High	49.5	6.9	
Ki-67 ≥ 2	Low	70.2	10.6	0.747	Low	75.5	9.8	0.919
	High	70.6	11.0		High	59.8	11.1	
Chemotherapy no	Low	133.1	6.1	0.019	Low	131.5	6.2	0.03
	High	72.9	9.3		High	58.0	7.6	
Chemotherapy yes	Low	101.7	8.9	0.379	Low	107.6	8.7	0.028
	High	64.4	10.7		High	48.9	8.6	
No endocrine therapy	Low	n.a. *		0.171	Low	85.3	12.5	0.861
	High	n.a. *			High	48.1	11.1	
Tamoxifen	Low	n.a. * n.a. *^§^		< 0.001 < 0.001^§^	Low	121.4 124.7^§^	7.7 7.6^§^	< 0.001 < 0.001^§^
	High	n.a. * n.a. *^§^			High	36.7 36.2^§^	6.3	
Aromatase Inhibitor	Low	n.a. * n.a. *^§^		0.108 0.122^§^	Low	111.0 108.8	8.1 9.0	0.53 0.463^§^
	High	n.a. * n.a. *^§^			High	95.7 95.7	6.0 6.0	

In multivariate Cox regression, we adjusted the hazard ratio (HR) of cytosolic and nuclear BCL3 for the parameters ER status and lymph node metastasis (Table 3). In both cases, BCL3 IRS turned out to be independent from these factors with an associated HR of about 1.8 and 2.9, respectively. Additionally, we adjusted nuclear for cytosolic BCL3-score by Cox regression analysis and found that the nuclear score was the predominant factor for relapse-free survival (HR = 2.5; CI: 1.35–4.57; p = 0.003). When we restricted this analysis to ER-positive cases treated with tamoxifen, the significance for cytosolic and nuclear BCL3-IRS increased even further (Table 3).

**Table 3:** Univariate and multivariate Cox regression survival analysis. Parameters BCL3 (either cytosolic or nuclear), estrogen receptor, HER2/Neu, and lymph node metastasis (N) were included (forward conditional) into the model. For multivariate Cox regression, the maximal *p* to be included into the model was set to 0.2. *HR*, hazard ratio. *Analysis restricted to ER-positive and tamoxifen-treated cases

Univariate cox regression			
Parameter	HR	95% CI	*p*
BCL3 cyt. *	1.853 5.095	1.002–3.428 2.281–11.381	0.049 < 0.001
BCL3 nucl. *	2.483 5.379	1.350–4.566 2.411–12.003	0.003 < 0.001
ER	0.654	0.396–1.081	0.098
HER2/NEU	1.700	1.034–2.795	0.036
N	1.841	1.174–2.887	0.008
Multivariate cox regression			
BCL3 cyt*	1.790 5.669	0.945–3.390 2.520–12.754	0.074 < 0.001
HER2/Neu*	1,716 2.867	0.902–3.268 1.272–6.462	0.100 0.011
N*	1.918 1.977	1.044–3.522 0.886–4.409	0.036 0.096
Multivariate cox regression			
BCL3 nuc. *	2.857 6,740	1.536–5.312 2.764–16.436	0.001 < 0.001
HER2/Neu*	2.014	0.889–4.562	> 0.2 0.093
N*	1.988 2.849	1.083–3.648 1.210–6.707	0.026 0.017

### Distribution of BCL3 mRNA in public BC datasets

**Fig. 6:**
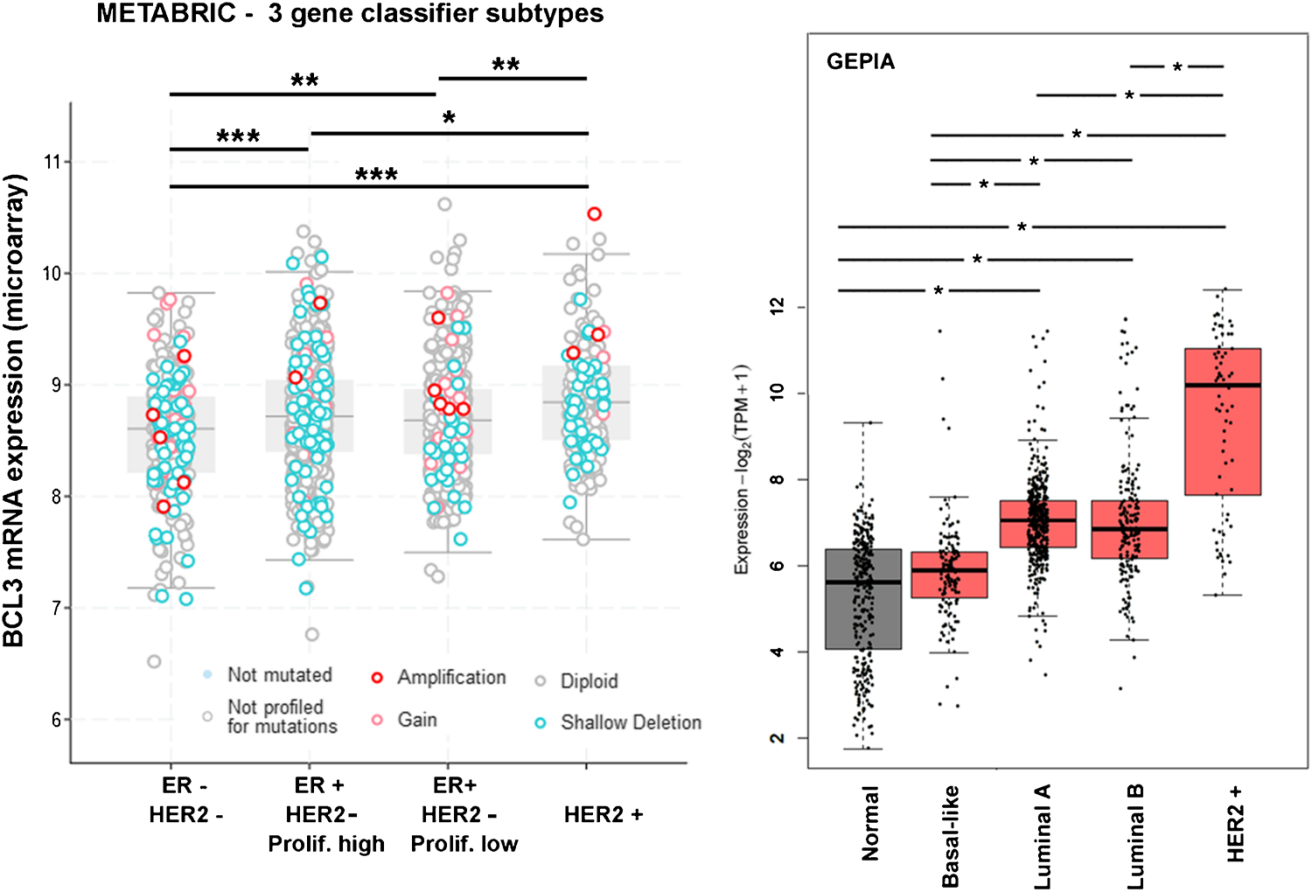
Distribution of BCL3 mRNA in public datasets. The METABRIC dataset was stratified according to the 3-gene classifier subtypes. Cases are labelled according to genomic BCL3 alterations such as mutations or copy number. Significance was determined with one-way ANOVA and Tamhane T2 post hoc analysis: *: *p* < 0.05, ** *p* < 0.01, *** *p* < 0.001. Results obtained from the GEPIA2 database are shown on the right

Additionally, we were interested in the distribution of *BCL3* mRNA abundance in a larger cohort of breast cancer cases. By using the GEPIA2 website [28], we found that BCL3 mRNA was more abundant in cancerous than in normal tissue with the exception of the basal subtype (Fig. 6). We additionally analyzed the gene expression data of the METABRIC cohort with respect to the 3-gene classifier subtypes, based upon ER- and HER2- as well as proliferation status (Fig. 6). It turned out for both datasets that HER2-over-expressing cases had the highest amounts of *BCL3* mRNA, whereas ER-/HER2-cases had the lowest abundance of this RNA. ER-positive cases ranged in between these two subtypes with no significant difference between low and high proliferating cases (luminal A and B).

## Discussion

Acquired tamoxifen or fulvestrant resistance is proposed to be at least in part a result of the interactions of BC cells with the tumor microenvironment. In vitro experiments suggested that this effect is mediated by the IGFBP5/BCL3 axis [22]. We were therefore interested to evaluate whether BCL3 could serve as a predictive biomarker for tamoxifen therapy success. Indeed, here we demonstrate a strong association of BCL3 abundance, determined by IHC, with the occurrence of a relapse under tamoxifen treatment. Most remarkable, there was no evidence for an association with the relapse-free survival of aromatase inhibitor–treated patients (Table 2). Nevertheless, the number of patients in this group was lower, which causes less statistical power.

Earlier studies already demonstrated that BCL3 is frequently overexpressed in breast cancer and mostly localized to the nucleus [29]. Based on these data, a potential role for p52 and BCL3 in breast cancer was postulated.

BCL3 protein abundance is regulated by an auto-regulatory loop via NF-kB [12]. Furthermore, the amount of BCL3 in the cytosol is determined by ubiquitinylation, which regulates its ongoing degradation [15]. In this localization, BCL3 has inhibitory functions on the NF-κB transcription factor, whereas upon activation of cells, BCL3 can be phosphorylated and located to the nucleus where it acts as a transcriptional coactivator. Our observation that BCL3 staining is present in cytosol and nucleus in varying amounts, suggests a functional difference of BCL3 in these tumor cells, especially a different activation status of the protein. This is supported by significant differences in the correlation of BCL3 cytosolic and nuclear IRS with clinico-pathological parameters.

The cytosolic abundance correlated with larger tumors (T > 2) and high proliferation (Ki-67 > 1 (Table 1), which is in line with the proposal that cytosolic BCL3 can act independently of NF-kB on proliferation and metastasis [13]. For example, the importance of BCL3 localization has been evaluated by Saamarthy et al. (2015) for colon cancer [17]. Here, the cytoplasmic localization was associated with high proliferation as indicated by Ki-67 status and negative for apoptosis markers, thus being important for tumor growth. However, in our breast cancer cohort, nuclear localized BCL3 seemed more important for RFS than the cytosolically localized protein. Nuclear abundance, which can be expected to represent activated BCL3, thus driving transcription as co-activator, did not correlate with most clinico-pathological factors. Both localizations, however, strongly correlated with the occurrence of a relapse under tamoxifen treatment.

The idea that nuclear localization is important for tamoxifen resistance is supported by our observation for the MCF-7 derived TamR cell lines (Fig. 1). Here, total BCL3 amount was reduced and predominantly localized to the nucleus. Similar data on the nuclear localization have been reported for fulvestrant-resistant MCF-7 sublines [30]. This would be consistent with a post-transcriptional activation of BCL3, resulting in increased degradation as well as translocation to the nucleus. Notably, the MCF7-TamR cell line also exhibited an altered behavior of NF-kB-signaling in response to toxic methylglyoxal [31]. This could well be interrelated with BCL3 amounts as it is a member of the IkB-family. It has also been shown that BCL3 is a regulator of c-Myc in MCF-7 cells [32]. In contrast to our observations on the protein abundance, BCL3 mRNA expression was slightly increased in TamR cells, as shown by our cDNA array experiments (logFc = 0.3, *p* = 0.03) [24]. Also our analysis of publicly available mRNA expression data showed no consistent correlation to our histochemistry protein data. For example, TNBC tumors did not show the significantly lower BCL3 protein levels as suggested by the mRNA data. This further suggests that BCL3 protein abundance is mostly the result of post-transcriptional regulation.

It is important to consider that our pathological study scored the BCL3 abundance before therapy had started. At this stage, BCL3 might be activated intrinsically or by interactions with the tumor micro-environment. Upon tamoxifen treatment, BCL3 may be activated by upstream signaling and then translocated to the nucleus. This can be especially relevant for tumors that already have high amounts of cytosolic BCL3 and could explain the development of tamoxifen resistance in these cases.

Interestingly, cytoplasmic BCL3 was significantly related to RFS in lobular carcinoma, whereas nuclear BCL3 was prognostic for ductal carcinoma as well. We suggest that this correlates with the role of cadherin signaling in lobular breast cancer. It is known from colorectal cancer that BCL3 promotes WNT-signaling and enhances β-catenin signaling [33]. In ductal breast cancer, β-catenin is intensively stained on the membrane, whereas in lobular carcinoma, the staining is described to be diffuse cytoplasmic or not detectable [34–36]. This holds for different functions of this molecule in the two entities: β-catenin can either act in cadherin-mediated cellular adhesion or in WNT-pathway-induced transcription. Interestingly, in our gene expression analysis of tamoxifen adapted MCF-7 cells [24], we also found the WNT pathway significantly altered under tamoxifen treatment (suppl. Figure 1). Consistently, the idea of a contribution of WNT signaling to tamoxifen adaption/resistance has been proposed by Ward et al. 2012 [37]. Furthermore, the WNT4 ligand was described to mediate endocrine resistance in lobular breast cancer cell lines [38]. Nevertheless, this idea needs further evaluation.

## Conclusions

Here we provide evidence for a contribution of BCL3 signaling in acquired tamoxifen resistance based upon a retrospective cohort analysis. BCL3-IRS might therefore become a valuable predictive biomarker for breast cancer.

## Supplementary Information

Below is the link to the electronic supplementary material.

Supplementary file1 (DOCX 275 KB)

## Data Availability

The datasets used and/or analyzed during the current study are available from the corresponding author on reasonable request.
