# Supplementary material for: BCL3 expression is strongly associated with the occurrence of breast cancer relapse under tamoxifen treatment in a retrospective cohort study
Source: Virchows Arch. 2022 Jan 12;480(3):529–41. doi: 10.1007/s00428-021-03238-8 (PMC8989858; doi:10.1007/s00428-021-03238-8)
Supplement: Supplementary file 1 — Supplementary file1 (DOCX 275 KB) [file 428_2021_3238_MOESM1_ESM.docx]

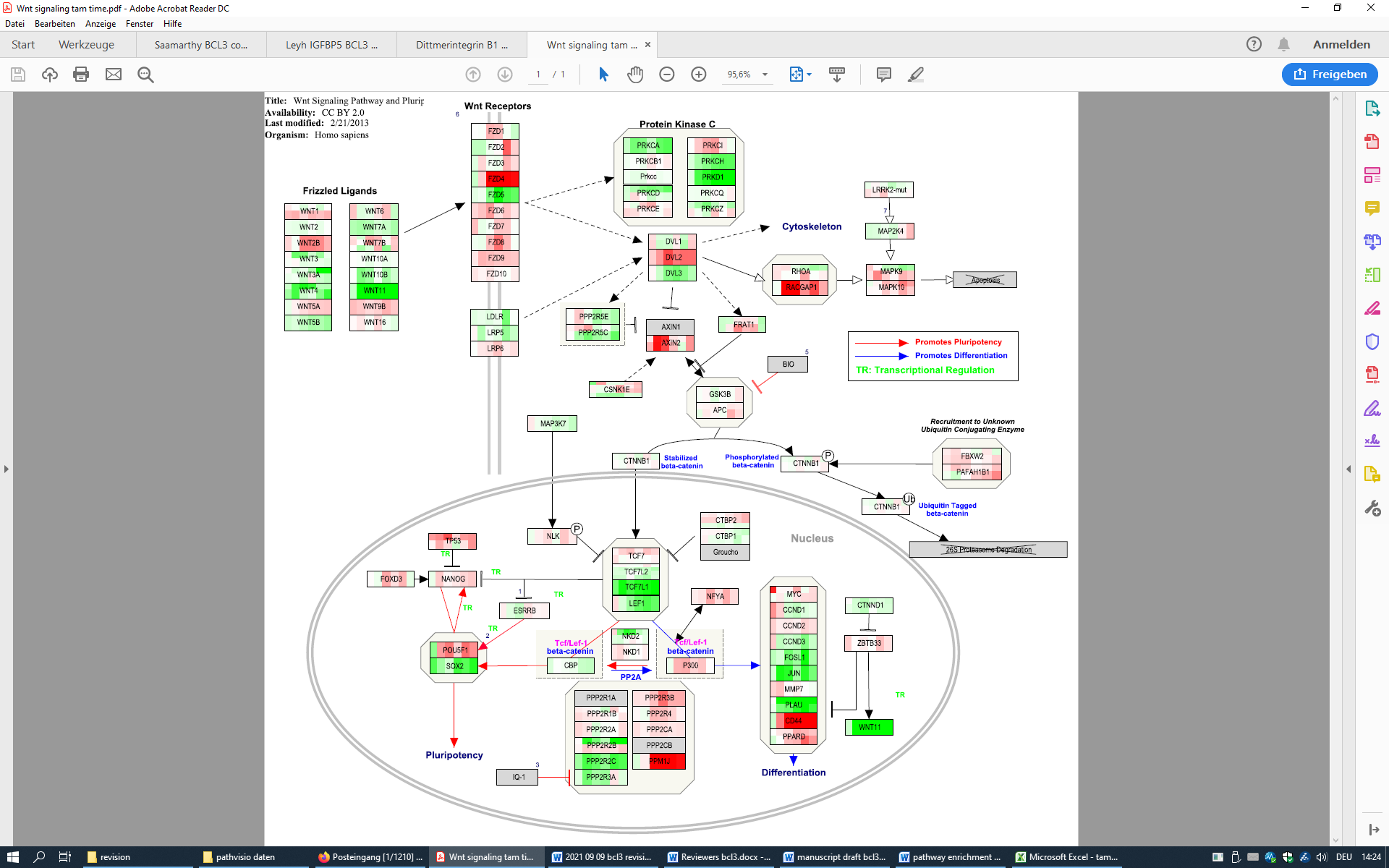


Fig. X: Pathway-enrichment analysis of tamoxifen regulated genes for components of the „WNT-Signalling pathway and pluripotency“. cDNA Array data were analysed using the pathvisio software. Genes used for pathway enrichment had to reach a log_2_Fc of at least 1 or -1 with an adjusted *p* < 0.01. This pathway reached a Z-score of 1.7 at the 12 week time point. Expression changes (log_2_Fc) of individual genes are indicated by colors: Green indicates enhanced and red indicates reduced transcript abundance at time points 24 h, 1, -2, 4-, 8- and 12 weeks
